# How Does the Sample Preparation of *Phytophthora infestans* Mycelium Affect the Quality of Isolated RNA?

**DOI:** 10.3390/cimb45040230

**Published:** 2023-04-18

**Authors:** Artemii A. Ivanov, Alexandr V. Tyapkin, Tatiana S. Golubeva

**Affiliations:** 1 Institute of Cytology and Genetics, Novosibirsk 630090, Russia; 2 Department of Natural Science, Novosibirsk State University, Novosibirsk 630090, Russia

**Keywords:** RNA isolation, *Phytophthora infestans*, oomycete

## Abstract

RNA isolation from fungi and fungus-like organisms is not an easy task. Active endogenous RNases quickly hydrolyze RNA after the sample collection, and the thick cell wall prevents inhibitors from penetrating the cells. Therefore, the initial collection and grinding steps may be crucial for the total RNA isolation from the mycelium. When isolating RNA from *Phytophthora infestans*, we varied the grinding time of the Tissue Lyser and used TRIzol and beta-mercaptoethanol to inhibit the RNase. In addition, we tested the mortar and pestle grinding of mycelium in liquid nitrogen, with this method showing the most consistent results. During the sample grinding with the Tissue Lyser device, adding an RNase inhibitor proved to be a prerequisite, and the best results were achieved using TRIzol. We considered ten different combinations of grinding conditions and isolation methods. The classical combination of a mortar and pestle, followed by TRIzol, has proved to be the most efficient.

## 1. Introduction

The isolation of RNA from fungi and fungus-like organisms appears to be a non-trivial task. Despite their external similarity, these organisms differ in physiology and often require individual approaches to supposedly routine procedures. Complications with RNA isolation are primarily due to the thick cell wall and active endogenous RNases, both issues having to be addressed at the earliest stages of the procedure, i.e., during cell grinding and lysis [1]. The success may depend on the isolation protocol [2,3], cultivation conditions [4], and even the material of the bead-beating system [1]. Most researchers favor grinding mycelium in liquid nitrogen [2,3,5,6], but this method is poorly scalable. However, automated processing of filamentous fungi and similar organisms might require adding a lysis buffer to the sample before grinding because it would inactivate the RNases [1]. Numerous studies focusing on the isolation of RNA from specific fungal species testify to the relevance of the need to optimize methods for dealing with specific organisms [3,5,7].

*Phytophthora infestans* is a parasitic oomycete affecting significant economic crops, such as potatoes and tomatoes. Though not related to true fungi, it exhibits a certain convergent similarity, forming a non-septic mycelium with a cell wall of cellulose and beta-glucans [8]. The difficulties encountered when isolating RNA from true fungi [1] are also relevant to *P. infestans*, but we could not find any specific publications on RNA isolation from this species. Those studies where the RNA isolated from oomycetes was used often reported TRIzol [9] or the RNEasy Plant Mini Kit [10] as the commonly known protocol without explanation. Given the above, the goal of the present work was to optimize the standard protocols specifically for the isolation/extraction of RNA from *P. infestans*.

To achieve this goal, we ground the mycelium using two methods: automatically on the Tissue Lyser LT with stainless steel beads and manually in a mortar with liquid nitrogen. We tested how effectively TRIzol and beta-mercaptoethanol inactivate the RNase and prevent RNA degradation during grinding. TRIzol (Thermo Fischer Scientific, Waltham, MA, USA), the RNEasy Plant Mini Kit (QIAGEN, Hilden, Germany), and their combination were attempted for RNA isolation after grinding. To assess the quality of the isolated RNA, we used the RNA integrity number (RIN) [11] and measured the concentration using a Qubit Fluorimeter (Thermo Fischer Scientific, Waltham, MA, USA).

## 2. Materials and Methods

### 2.1. Cultivation and Collection of Mycelium

Mycelium of *P. infestans* strain VZR18 was obtained from the All-Russian Institute of Plant Protection and grown on Petri dishes with Rye B medium [12] for 6 weeks in the dark at +14 °C. In addition, 3 samples from B_TQ-15 and MP_T each (Table 1) were grown for 8 weeks under the same conditions. Mycelium was collected with a dissecting needle. The mycelium collected was packed into 100 mg units and immediately placed in liquid nitrogen. RNA isolation was performed on the same day. In total, 33 samples were collected for the study.

For each of the 10 grinding and extraction techniques listed in Table 1, a total of 3 mycelium units were sampled. At least two different Petri dishes were used to harvest *P. infestans* for each technique. For the NB_T-15 and B_T-15 methods, the number of samples was reduced because of the insufficient amount of mycelium material in the relevant Petri dishes, with one sample of B_TQ-15 excluded because of suspected contamination.

After receiving the interim data, the RNA extraction was repeated with 3 more mycelium samples for the MP_T and B_TQ-15 methods. 

### 2.2. Grinding of Mycelium

Different techniques and parameters of mycelium grinding were attempted during the experiment:

1. With a mortar and pestle in liquid nitrogen.
2. The frozen material was subjected to grinding using Tissue Lyser LT (QIAGEN, Hilden, Germany): oscillation frequency of 50 Hz, processing times of 15 s, 30 s, and 1 min, bead material–stainless steel. The rotor was cooled down to −70 °C. Immediately after grinding, 1 mL TRIzol (Thermo Fischer Scientific, Waltham, MA, USA) or 450 μL RLT buffer (QIAGEN, Hilden, Germany) with beta-mercaptoethanol for RNase inhibition was added to the samples.
3. Similar to point 2, but with TRIzol and RLT buffer (RNEasy Plant Mini Kit, QIAGEN, Hilden, Germany) added before grinding.

All parameter combinations used with the number of samples are shown in Table 1.

After grinding and adding the lysis agent, the samples were thoroughly mixed and incubated on ice for 10 min.

### 2.3. RNA Isolation

Three different methods were attempted for RNA isolation:

1. A standard TRIzol protocol according to the manufacturer’s recommendations [13].
2. RNEasy Plant Mini Kit (QIAGEN, Hilden, Germany) according to the manufacturer’s protocol.
3. A combination of TRIzol and RNEasy spin column (QIAGEN, Hilden, Germany) methods.

For each method, RNA elution was performed with 50 uL of RNase-purified water. The aliquots for the RIN determination and concentration measurements were selected immediately after isolation. The RNA was stored at −80 °C. A complete protocol for the combined TRIzol and spin column RNA isolation is given in Table 2.

### 2.4. Evaluation of the Amount and Quality of Isolated RNA

The concentration of isolated RNA was measured using a Qubit 4 Fluorimeter (Thermo Fisher Scientific, Waltham, MA, USA) with an HS RNA Assay kit (Thermo Fisher Scientific, Waltham, MA, USA). A 2100 Bioanalyzer (Agilent Technologies, Santa Clara, CA, USA) and RNA 6000 pico chip (Agilent Technologies, Santa Clara, CA, USA) were used to assess the integrity and the length distribution of isolated RNA. The analysis was carried out in the Genomics Center at the Institute of Chemical Biology and Fundamental Medicine, Siberian Branch of the Russian Academy of Sciences (Novosibirsk, Russia). The 2100 Expert Agilent software (Agilent Technologies, Santa Clara, CA, USA) uses an algorithm to evaluate RNA integrity based on capillary electrophoresis data with a specific dye. As a result, the sample is assigned an RNA integrity number (RIN) ranging from 1 to 10, with 1 representing completely degraded RNA [11].

### 2.5. Statistical Analysis

For each group of experimental conditions described above (Table 1), we performed a statistical analysis and data visualization in R (R Core Team, 2013; version 4.1.2), using *car* [14] and *ggpubr* R packages. We used one-way ANOVA to measure the effect of experimental conditions on the RIN and the concentration of extracted RNA. Pairwise comparisons between groups were conducted using a *t*-test (*p*-value threshold <0.05).

## 3. Results

### 3.1. Evaluation of Isolated RNA Quality

**Figure 1:**
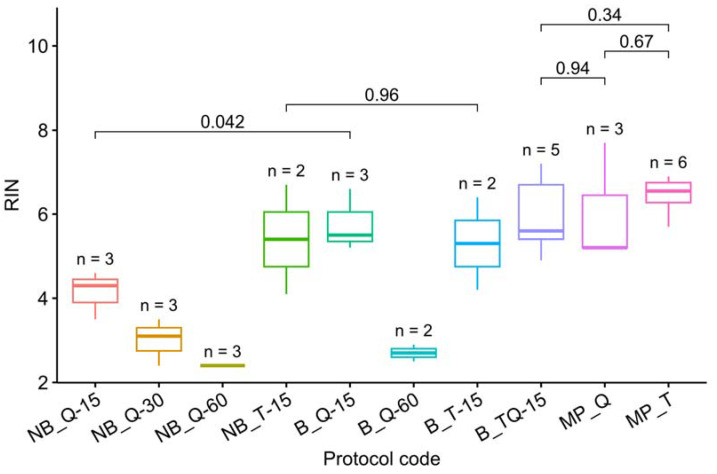
RIN visualization. The letters up to “_” denote the grinding method: MP–mortar and pestle, NB–Tissue Lyser without buffer, and B–with buffer. The following letters denote the isolation protocol: T–TRIzol, Q–RNEasy Plant Mini Kit, and TQ–a combination (Table 1). The number after “-” denotes the grinding time on the Tissue Lyser in seconds. The letter *n* marks the number of samples used for each method. The *t*-test *p*-values are given for each pair of methods discussed in the text. The RIN of samples processed on Tissue Lyser without buffer (except NB_T-15) or over 15 s was found to be statistically significantly lower than that of the other samples (*t*-test, *p* = 3.6 × 10^−9^ < 0.05). The omission of NB_T-15 from this list may indicate that, with a short crushing time (15 s), the bulk of the RNA degrades after the Tissue Lyser grinding. Thus, TRIzol appears to be a more effective preservative than beta-mercaptoethanol, thwarting the degradation even if added with a delay (Figure 1).

The RIN measurements for all protocols are presented in Table 3 and visualized in Figure 1. Note that grinding with a mortar and pestle is the best way to prevent RNA degradation. In the case of grinding with steel beads on a Tissue Lyser LT, RNA was preserved only by adding an RNase inhibiting buffer and only with a minimum grinding time of 15 s. 

We have not succeeded in finding statistically significant differences in the RIN between the three best techniques (Figure 1), but the combination of TRIzol and manual crushing in liquid nitrogen (MP_T) provided a smaller discrepancy in the results.

**Figure 2:**
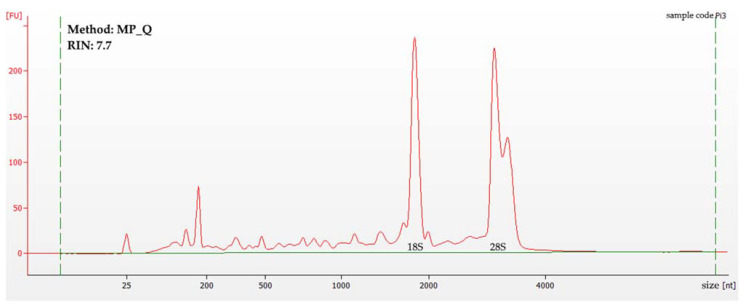
A sample of the 2100 Bioanalyzer data from a 6000 RNA pico chip. The *X*-axis represents the molecule size in nucleotides, with the *Y*-axis representing the fluorescence in fluorescence units. The green dotted lines are thresholds. The peaks for 18S and 28S rRNA are marked.

An example of the 2100 Bioanalyzer data obtained during the experiment can be found in Figure 2. For this particular sample, the method of RNA isolation was a mortar and pestle grinding in liquid nitrogen, followed by RNEasy Plant Mini Kit RNA extraction (MP_Q).

### 3.2. RNA Concentration Measurements

**Figure 3:**
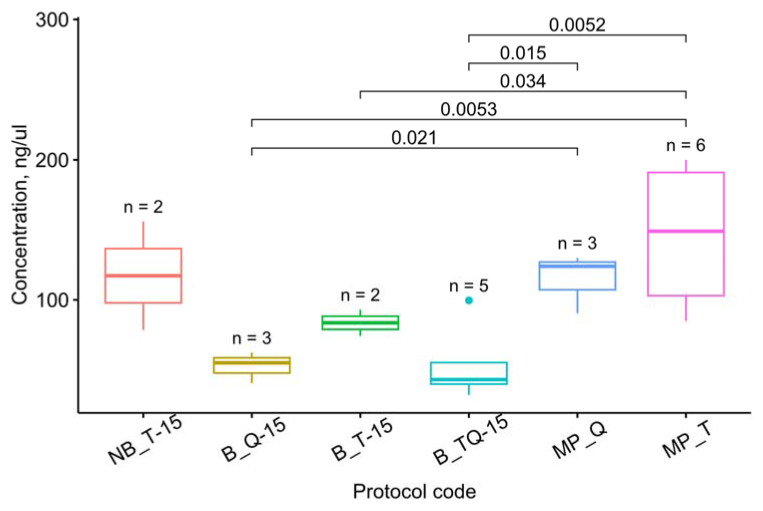
Concentrations for the methods that yield the average RIN values of >5. The letters up to “_” denote the grinding method: MP–mortar and pestle, NB–Tissue Lyser without buffer, and B–with buffer. The following letters denote the isolation protocol: T–TRIzol, Q–RNEasy Plant Mini Kit, and TQ–a combination (Table 1). The number after “-” denotes the grinding time on the Tissue Lyser in seconds. The letter *n* marks the number of samples used for each method. The *t*-test *p*-values are given for each pair of methods discussed in the text. Dots represent outliers.

The concentration measurement results are averaged in Table 2. Figure 3 visualizes the concentrations for the methods yielding the average RIN values of >5. This threshold was set to exclude such methods as NB_Q-60 and B_Q-60, which produced decent amounts of completely degraded RNA and could therefore disturb the observer.

**Figure 4:**
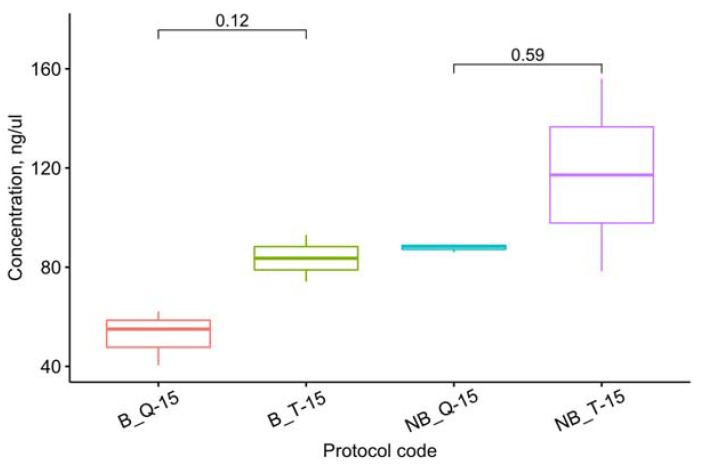
Effects of the extraction method on the RNA concentration of the samples ground in the buffer for 15 s. The letters up to “_” denote the grinding method: MP–mortar and pestle, NB–Tissue Lyser without buffer, and B–with buffer. The following letters denote the isolation protocol: T–TRIzol, Q–RNEasy Plant Mini Kit, and TQ–a combination (Table 1). The number after “-” denotes the grinding time on the Tissue Lyser in seconds. The *t*-test *p*-values are given for each pair of methods discussed in the text.

Among the methods mentioned above, the highest amount of RNA was isolated with a mortar and pestle. Despite the apparently greater efficiency of TRIzol compared with the RNEasy Plant Mini Kit when processed on the Tissue Lyser, the one-way ANOVA suggests these differences are not statistically significant (F(3, 6) = 3.02, *p* = 0.116), as shown in Figure 4. 

**Figure 5:**
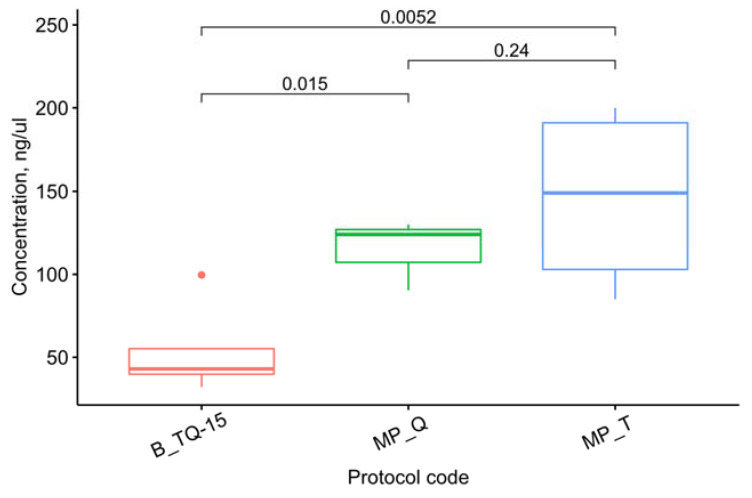
Pairwise comparison of the concentrations obtained by the three methods with the highest average RIN. The letters up to “_” denote the grinding method: MP–mortar and pestle, NB–Tissue Lyser without buffer, and B–with buffer. The following letters denote the isolation protocol: T–TRIzol, Q–RNEasy Plant Mini Kit, and TQ–a combination (Table 1). The number after “-” denotes the grinding time on the Tissue Lyser in seconds. The *t*-test *p*-values are given for each pair of methods. Dots represent outliers.

Thus, the RNEasy Plant Mini Kit can provide a concentration of RNA isolated from *P. infestans* comparable to TRIzol: when using a mortar and pestle, this method proves equally efficient (Figure 5).

## 4. Discussion

Given the results obtained, a mortar and pestle method can be considered to be the best choice. This method provides cooling in liquid nitrogen throughout the grinding process and has been used by default in works for the isolation of very high-quality RNA from fungi [2,3]. Nevertheless, the low productivity of the mortar and pestle grinding limits the possibility of scaling. The Tissue Lyser can be used for this purpose: with a short crushing time and the obligatory application of an RNase inhibitor (preferably TRIzol), its performance is not much worse. Fifteen seconds proves to be enough to grind *Phytophthora*. At the same time, in contrast to the data [1], beta-mercaptoethanol turned out to be unable to protect the RNA for a longer period of time. The difference is likely because Leite et al. isolated RNA from a more thermophilic organism, while a slight increase in temperature is sufficient to activate *P. infestans* RNases.

In this study [2], TRIzol and grinding in liquid nitrogen provided a mean RIN = 6.2, which is similar to our results. However, adding glycogen and the purification step through a 26G needle led to a significant increase in RNA integrity up to a mean RIN = 9.5. Of no less interest are the results of protocols 4 and 5 from the same study: purification on the RNEasy column of isolated TRIzol RNA also led to a significant increase in the RIN. The additional purification on the column seems to remove some of the degraded RNA.

However, the RNA with an RIN ranging from 6 to 7 can also be used for library formation [3]. In particular, BGI accepts such samples for sequencing [15]. This RNA can also be used for other molecular genetic applications, such as quantitative PCR, which is less demanding on the RIN but is often used as a quality standard [5,16].

One of the reasons for the reduced integrity of isolated RNA could be the long process of collecting mycelium from the nutrient medium, which is associated with considerable damage. Therefore, using alternative cultivation techniques, such as those in [4,6,16], may help to improve the results.

This work allowed us to determine the most promising approaches to mycelial grinding and RNA isolation from *P. infestans*. Thus, grinding mycelium with a mortar and pestle in liquid nitrogen is preferable both in terms of the RIN and the concentration of isolated RNA. If it is necessary to scale up the experiment, it is acceptable to use a bead-beating system, with the mandatory addition of lysis buffer before grinding, as well as minimization of the exposure time.

## Figures and Tables

**Table 1:** The scheme of the experiment.

Grinding Method	Tissue Lyser								Mortar & Pestle	
Grinding time	15 s					30 s	60 s		-	
Grinding in buffer	No		Yes			No	No	Yes	No	
Extraction protocol	TRIzol	Qiagen	Qiagen + TRIzol	Qiagen	TRIzol	Qiagen	Qiagen	Qiagen	TRIzol	Qiagen
Protocol code	NB_T-15	NB_Q-15	B_TQ-15	B_Q-15	B_T-15	NB_Q-30	NB_Q-60	B_Q-60	MP_T	MP_Q
Number of technical replicates	2	3	5	3	2	3	3	3	6	3

The letters up to “_” denote the grinding method: MP–mortar and pestle, NB–Tissue Lyser without buffer, and B–with buffer. The following letters denote the isolation protocol: T–TRIzol, Q–RNEasy plant mini kit, and TQ–a combination (Table 2). The number after “-” denotes the grinding time on the Tissue Lyser in seconds.

**Table 2:** Combined TRIzol and spin column RNA isolation protocol.

TRIzol + RNEasy Spin Column Protocol
Homogenize the sample in TRIzol (1 mL of TRIzol per 100 mg of tissue)
2. Incubate for 10 min on ice.
3. Add 0.2 V of chloroform to each tube.
4. Stir by turning the tubes for 30 s, then vortex for 1–2 s. Incubate for 2 min on ice.
5. Centrifuge for 15 min, 15,000× *g*, 4 °C
6. Carefully transfer the aqueous phase to a new microcentrifuge tube, add 0.5 V of 96% ethanol, and mix by pipetting.
Then the RNEasy Plant Mini Kit is used according to the manufacturer protocol.

**Table 3:** Mean concentrations and RINs.

Protocol Code	Mean RIN ± SEM	Mean C ± SEM (ng/uL)
NB_Q-15	4.13 ± 0.33	87.87 ± 0.96
NB_Q-30	3.00 ± 0.32	39.93 ± 15.34
NB_Q-60	2.40 ± 0.00	114.20 ± 19.10
NB_T-15	5.40 ± 1.30	117.20 ± 38.80
B_Q-15	5.77 ± 0.43	52.53 ± 6.41
B_Q-60	2.70 ± 0.20	135.00 ± 3.00
B_T-15	5.30 ± 1.10	83.60 ± 9.40
B_TQ-15	5.96 ± 0.43	54.00 ± 11.98
MP_Q	6.03 ± 0.83	114.80 ± 12.32
MP_T	6.45 ± 0.18	145.93 ± 20.71

The letters up to “_” denote the grinding method: MP–mortar and pestle, NB–Tissue Lyser without buffer, and B–with buffer. The following letters denote the isolation protocol: T–TRIzol, Q–RNEasy plant mini kit, and TQ–a combination (Table 1). The number after “-” denotes the grinding time on the Tissue Lyser in seconds. The one-way ANOVA suggests statistically significant differences between RINs (F(9, 22) = 9.73, *p* = 7.57 × 10^−6^) and concentrations (F(9, 22) = 4.68, *p* = 0.00151).

## Data Availability

Not applicable.
